# Angiotensin I-Converting Enzyme Inhibitory Peptides of Chia (*Salvia hispanica*) Produced by Enzymatic Hydrolysis

**DOI:** 10.1155/2013/158482

**Published:** 2013-05-21

**Authors:** Maira Rubi Segura Campos, Fanny Peralta González, Luis Chel Guerrero, David Betancur Ancona

**Affiliations:** Facultad de Ingeniería Química, Universidad Autónoma de Yucatán, Periférico Norte. Km 33.5, Tablaje Catastral 13615, Colonia Chuburná de Hidalgo Inn, 97203 Mérida, YUC, Mexico

## Abstract

Synthetic angiotensin I-converting enzyme (ACE-I) inhibitors can have undesirable side effects, while natural inhibitors have no side effects and are potential nutraceuticals. A protein-rich fraction from chia (*Salvia hispanica* L.) seed was hydrolyzed with an Alcalase-Flavourzyme sequential system and the hydrolysate ultrafiltered through four molecular weight cut-off membranes (1 kDa, 3 kDa, 5 kDa, and 10 kDa). ACE-I inhibitory activity was quantified in the hydrolysate and ultrafiltered fractions. The hydrolysate was extensive (DH = 51.64%) and had 58.46% ACE-inhibitory activity. Inhibition ranged from 53.84% to 69.31% in the five ultrafiltered fractions and was highest in the <1 kDa fraction (69.31%). This fraction's amino acid composition was identified and then it was purified by gel filtration chromatography and ACE-I inhibition measured in the purified fractions. Amino acid composition suggested that hydrophobic residues contributed substantially to chia peptide ACE-I inhibitory strength, probably by blocking angiotensin II production. Inhibitory activity ranged from 48.41% to 62.58% in the purified fractions, but fraction F1 (1.5–2.5 kDa) exhibited the highest inhibition (IC_50_ = 3.97 *μ*g/mL; 427–455 mL elution volume). The results point out the possibility of obtaining bioactive peptides from chia proteins by means of a controlled protein hydrolysis using Alcalase-Flavourzyme sequentional system.

## 1. Introduction

Cardiovascular disease (CVD) affects the heart and blood vessels and is the principal cause of death worldwide. Considered the primary risk factor for CVD, high blood pressure, or hypertension, consists of a sustained increase in blood pressure levels. In 2000, >25% of the population worldwide (approximately 1 billion) suffered from hypertension, a figure predicted to increase to 1.56 billion by 2025 [1].

Angiotensin I-converting enzyme (peptidyl carboxy peptidase, EC 3.4.15.1, ACE) belongs to the class of zinc proteases that require zinc and chloride for activation. ACE plays an important role in blood pressure regulation via the renin-angiotensin system (RAS) and the kallikrein-kinnin system (KKS). In the KKS, ACE inactivates the vasodilator bradykinin, while in the RAS, ACE acts as an exopeptidase cleaving His-Leu from the C-terminal of decapeptide angiotensin I and producing the potent vasoconstrictor octapeptide angiotensin II [2].

Use of enzyme technologies for protein recovery and modification has led to production of a broad spectrum of food ingredients and industrial products. Hydrolysis selectivity is commonly manipulated by employing proteases from different sources due to their specificity for peptide bonds adjacent to certain amino acids. Enzymatic hydrolysis of food proteins is an efficient way to recover potent bioactive peptides [3]. ACE-I inhibitory peptides derived from food proteins have attracted particular attention for their ability to prevent hypertension. Compared with chemosynthetic drugs, peptides from food proteins may have reduced toxic effects in humans. They therefore hold promise as potent functional food additives and represent a healthier and more natural alternative to ACE inhibitor drugs. Dietary ACE-I inhibitory peptides may be bioavailable. Some ACE-I inhibitory peptides resist digestion, can be absorbed in the intestine, and are stable in the blood, suggesting they may produce an acute blood pressure-lowering effect after oral administration. The first ACE-I inhibitory peptide was isolated from snake venom [4], and since then many others have been discovered in enzymatic hydrolysates of different food proteins. Food protein sources used to date include casein, whey protein, fish protein, chicken eggs, and wheat germ. Inhibitory activities and sequences are remarkably different in many of these peptides [5]. The soybean proteins are other novel sources to production of the ACE inhibitory peptides. *β*-conglycinin and glycinin were hydrolysed by an acid proteinase from *Monascus purpureus*. The degree of hydrolysis and inhibitory activities of angiotensin I-converting enzyme (ACE) increased with increasing proteolysis time [6].

Population growth and shrinking food resources are ongoing challenges in developing countries, while excessive animal protein intake and associated unhealthy levels of saturated fats exposure are increasingly common in developed countries. In response, research interest has steadily grown in the search for new sources of proteins from nonconventional raw materials. The genus *Salvia* L. belongs to the Lamiaceae family and includes about 900 species found worldwide, most mainly in the Mediterranean, Southeast Africa, and Central and South America. Cultivated for culinary, medicinal, and ornamental uses, *Salvia* species form part of ethnopharmacological traditions and are an important crop, especially for small farmers [7]. Recent research has addressed the chemical composition, biological properties, and possible applications of its essential oils, which may be sources for economically promising natural products with uses in the food, pharmaceutical, and cosmetic industries. The present study objective was to identify and quantify ACE-I inhibitory activity in protein hydrolysates from a *Salvia hispanica *protein rich fraction hydrolyzed with an Alcalase-Flavourzyme sequential system, and in ultrafiltered fractions from this hydrolysate.

## 2. Materials and Methods

### 2.1. Materials

Chia (*S. hispanica*, L.) seeds were obtained in Yucatan state, Mexico. Reagents were of analytical grade and purchased from J. T. Baker (Phillipsburg, NJ, USA), Sigma (Sigma Chemical Co., St. Louis, MO, USA), Merck (Darmstadt, Germany), and Bio-Rad (Bio-Rad Laboratories Inc., Hercules, CA, USA). Angiotensin-converting enzyme from rabbit lung (2 units/mg protein) was purchased by Sigma (A6778 Sigma Chemical Co., St. Louis, MO, USA). The Alcalase 2.4 L and Flavourzyme 500 MG enzymes were purchased from Novo Laboratories (Copenhagen, Denmark). 

### 2.2. Protein Rich Fraction

Flour was produced from 6 kg chia seed by first removing all impurities and damaged seeds, crushing the remaining sound seeds (Moulinex DPA139, Zapopan, Mexico), and milling them (Krups 203 Mill, Mexico City, Mexico). Oil extraction from the milled seeds was done with hexane in a Soxhlet system for 2 h. The remaining fraction was milled with 0.5 mm screen (Thomas Model 4 Wiley, Swedesboro, NJ, USA). The defatted chia flour was dried in a Labline stove at 60°C for 24 h. Extraction of the protein-rich fraction was done by dry fractionation of the defatted flour according to Vázquez-Ovando et al. [8]. Briefly, 500 g flour was sifted for 20 min using a Tyler 100 mesh (140 *μ*m screen) and a Ro-Tap agitation system. 

### 2.3. Enzymatic Hydrolysis

The chia protein-rich fraction was hydrolyzed in batches by sequential treatment with Alcalase and Flavourzyme. A predigestion with Alcalase for 60 min was followed by incubation with Flavourzyme for 90 min. Hydrolysis conditions were substrate concentration, 2 g/100 g; enzyme/substrate ratio, 0.3 AU g^−1^ for Alcalase and 50 LAPU g^−1^ for Flavourzyme; pH, 7 for Alcalase and 8 for Flavourzyme and temperature, 50°C. Hydrolysis was done in a reaction vessel equipped with a stirrer, thermometer, and pH electrode. In all treatments, the reaction was stopped by heating to 85°C for 15 min, followed by centrifuging at 9880 ×g for 20 min to remove the insoluble portion [9].

### 2.4. Degree of Hydrolysis

Degree of hydrolysis (DH) was calculated by determining free amino groups with o-phthaldialdehyde following the methodology described by Nielsen et al. [10] as follows: DH = *h*/*h*_tot_∗100, where *h*_tot_ is the total number of peptide bonds per protein equivalent, and *h* is the number of hydrolyzed bonds. The *h*_tot_ factor is dependent on the raw material amino acid composition and was determined by reverse-phase high performance liquid chromatography (RP-HPLC) [11]. Samples (2–4 mg protein) were treated with 4 mL of 6 mol equivalent to L^−1^ HCl, placed in hydrolysis tubes, and gassed with nitrogen at 110°C for 24 h. They were then dried in a rotavapor and suspended in 1 mol L^−1^ sodium borate buffer at pH 9.0. Amino acid derivatization was performed at 50°C using diethyl ethoxymethylenemalonate. Amino acids were separated using HPLC with a reversed-phase column (300 × 3.9 mm, Nova-Pak C18, 4 mm; Waters) and a binary gradient system with 25 mmol L^−1^ sodium acetate containing (A) 0.02 g L^−1^ sodium azide at pH 6.0 and (B) acetonitrile as solvent. Flow rate was 0.9 mL min^−1^, and elution gradient was time 0.0–3.0 min, linear gradient A : B (91 : 9) to A–B (86 : 14); time 3.0–13.0 min, elution with A–B (86–14); time 13.0–30.0 min, linear gradient A–B (86 : 14) to A–B (69 : 31); time 30.0–35.0 min, elution with A–B (69 : 31).

### 2.5. Hydrolysate Fractionation by Ultrafiltration

The hydrolysate was fractionated by ultrafiltration [12], using a high performance ultrafiltration cell (Model 2000, Millipore). Five fractions were prepared using four molecular weight cut-off (MWCO) membranes: 1 kDa, 3 kDa, 5 kDa, and 10 kDa. Soluble fractions prepared by centrifugation (9880 ×g for 20 min) were passed through the membrane starting with the largest MWCO membrane cartridge (10 kDa). Retentate and permeate were collected separately, and the retentate recirculated into the feed until maximum permeate yield was reached at this size, as indicated by a decrease in permeate flow rate. Permeate from the 10 kDa membrane was then filtered through the 5 kDa membrane with recirculation until maximum permeate yield was reached. The 5 kDa permeate was then recirculated through the 3 kDa membrane and the 3 kDa permeate through the 1 kDa membrane. This process minimized contamination of the larger molecular weight fractions with smaller molecular weight fractions, while producing enough retentates and permeates for the following analyses. The five ultrafiltered peptide fractions (UPF) were prepared and designated as >10 kDa (10 kDa retentate); 5–10 kDa (10 kDa permeate–5 kDa retentate); 3–5 kDa (5 kDa permeate–3 kDa retentate); 1–3 kDa (3 kDa permeate–1 kDa retentate); <1 kDa (1 kDa permeate). 

### 2.6. ACE-I Inhibitory Activity

ACE-I inhibitory activity in the hydrolysate and its UPF was analyzed following the method of Hayakari et al. [13] which is based on the fact that ACE-I hydrolyzes hippuryl-L-histidyl-L-leucine (HHL) yielding hippuric acid and L-histidyl-L-leucine. This method relies on the colorimetric reaction of hippuric acid with 2,4,6-trichloro-S-triazine (TT) in a 0.5 mL incubation mixture containing 40 *μ*mol potassium phosphate buffer (pH 8.3), 300 *μ*mol sodium chloride, 40 *μ*mol 3% HHL in potassium phosphate buffer (pH 8.3), and 100 mU/mL ACE-I. This mixture was incubated at 37°C/45 min and the reaction terminated by addition of TT (3% v/v) in dioxane and 3 mL 0.2 M potassium phosphate buffer (pH 8.3). After centrifuging the reaction mixture at 10,000 ×g for 10 min, enzymatic activity was determined in the supernatant by measuring absorbance at 382 nm. All runs were done in triplicate. ACE-I inhibitory activity was quantified by a regression analysis of ACE-I inhibitory activity (%) versus peptide concentration, and IC_50_ values (i.e., the peptide concentration in *μ*g protein/mL required to produce 50% ACE-I inhibition under the described conditions) was defined and calculated as follows:

(1) $$\begin{array}{c} \text{ACE-I inhibitory activity}\;\left(\%\right)=\frac{\left(A-B\right)}{\left(A-C\right)}\times 100, \end{array}$$

where *A* represents absorbance in the presence of ACE-I sample, *B* absorbance of the control, and *C* absorbance of the reaction blank.

Consider the following:

(2) $$\begin{array}{c} {\text{IC}}_{50}=\frac{\left(50-b\right)}{m}, \end{array}$$

where *b* is the intersection and *m* is the slope. 

### 2.7. Amino Acid Composition

Amino acid composition was determined in the UPF with the highest biological activity, according to the method of Alaiz et al. [11] Samples (2–4 mg protein) were treated with 4 mL of 6 mol equivalent L^−1^ HCl, placed in hydrolysis tubes, and gassed with nitrogen at 110°C for 24 h. They were then dried in a rotavapor and suspended in 1 mol L^−1^ sodium borate buffer at pH 9.0. Amino acid derivatization was performed at 50°C using diethyl ethoxymethylenemalonate. Amino acids were separated using HPLC with a reversed-phase column (300 × 3.9 mm, Nova-Pak C18, 4 mm; Waters) and a binary gradient system with 25 mmol L^−1^ sodium acetate containing (A) 0.02 g L^−1^ sodium azide at pH 6.0 and (B) acetonitrile as solvent. Flow rate was 0.9 mL min^−1^, and the elution gradient was time 0.0–3.0 min, linear gradient A : B (91 : 9) to A–B (86 : 14); time 3.0–13.0 min, elution with A–B (86–14); time 13.0–30.0 min, linear gradient A–B (86 : 14) to A–B (69 : 31); time 30.0–35.0 min, elution with A–B (69 : 31).

### 2.8. G-50 Gel Filtration Chromatography

After filtration through 10, 5, 3, and 1 kDa membranes in a high performance ultrafiltration cell, 10 mL of the fraction with highest ACE-I inhibitory activity was injected into a Sephadex G-50 gel filtration column (3 cm × 79 cm) at a flow rate of 25 mL/h of 50 mM ammonium bicarbonate (pH 9.1). The resulting fractions were collected to assay ACE-I inhibitory activity [13]. Peptide molecular masses were determined by referring to a calibration curve running molecular mass markers on the Sephadex G-50 under identical conditions and those used for the test samples. Molecular mass standards were thyroglobulin (670 kDa), bovine gamma globulin (158 kDa), equine myoglobin (17 kDa), vitamin B_12_ (1.35 kDa), and Thr-Gln (0.25 kDa). Fractions selected for further peptide purification were pooled and lyophilized before RP-HPLC. 

### 2.9. Statistical Analysis

All results were analyzed in triplicate using descriptive statistics to estimate means and variation. One-way ANOVAs were run to evaluate *in vitro* ACE-I inhibitory activity and a Duncan multiple range done to identify differences between treatments. All analyses were done according to Montgomery [14] and processed using the Statgraphics Plus version 5.1 software.

## 3. Results and Discussion

### 3.1. Enzymatic Hydrolysis of Protein-Rich Fraction

With a protein content of 46.7%, the chia protein-rich fraction proved to be good starter material for hydrolysis. Production of extensive (i.e., >50% DH) hydrolysates requires use of more than one protease because a single enzyme cannot achieve such high DHs within a reasonable time period. For this reason, an Alcalase-Flavourzyme sequential system was used in the present study to produce an extensive hydrolysate. Alcalase (EC 3.4.21.62) is a proteinase from *Bacillus licheniformis* and the Flavourzyme (EC 3.4.11.1) is a fungal protease from *Aspergillus oryzae* with both endo- and exopeptidase activities. The bacterial endoprotease Alcalase is limited by its specificity, resulting in DHs no higher than 20%–25%, depending on substrate, but can attain these DHs in a relatively short time under moderate conditions. When Alcalase is used to hydrolyze protein it tends to produce peptides whose C-terminals are amino acids with large side chains and no charge (aromatic and aliphatic amino acids), such as Ile, Leu, Val, Met, Phe, Tyr, and Trp. The hydrolysis process is accelerated when peptide N-terminals have hydrophobic amino acids [15]. The fungal protease Flavourzyme has broader specificity, which, when combined with its exopeptidase activity, can generate DH values as high as 50%. Flavourzyme is recommended for production of hydrolysates or peptides with biological activity and low bitterness. Both Alcalase and Flavourzyme tend to generate peptides with hydrophobic amino acid C-terminals, and QSAR analyses of ACE-I inhibitory peptides have shown that peptides with hydrophobic amino acid C-terminals exhibit potentially strong ACE-I inhibition. Therefore, Alcalase and Flavourzyme are probably suitable for preparing high-activity ACE-I inhibiting peptides. In addition, both proteases are suitable for industrial applications and are microbial enzymes, meaning that they are easily obtained and relatively low cost compared to other enzymes such as proteinase K and chymotrypsin C [15].

Chia hydrolysates obtained with the Alcalase-Flavourzyme sequential system had clear biological activity and are promising prospects for use in new product development. The highest DH in the present study (51.6%) was attained with Flavourzyme at 150 min. However, it was made possible by Alcalase predigestion, which increases the number of N-terminal sites, thus facilitating hydrolysis by Flavourzyme. This DH was higher than reported for hard-to-cook bean hydrolyzed with Alcalase-Flavourzyme (43%) or pepsin-pancreatin (26.2%) for 90 min [16]. But it was lower than reported for chickpea (65%) hydrolyzed with Alcalase-Flavourzyme at 150 min [17] and for rapeseed (60%) hydrolyzed with the same system at 3 h [18]. Variations in DH values are probably the result of protein source and hydrolytic specificity. When hydrolyzed sequentially with Alcalase and Flavourzyme, chia *S. hispanica *is an appropriate substrate for producing extensive hydrolysates (DH higher than 10%), and a natural source of peptides with potential bioactivity.

### 3.2. ACE-I Inhibitory Activity

A number of natural ACE-I inhibitors have been isolated from different organism proteins, including peptides extracted by enzymatic hydrolysis. When added to food systems, enzymatic hydrolysates have exhibited advantages such as improved water-binding capacity, emulsifying stability, protein solubility, and nutritional quality. Enzymatic hydrolysis has become a valuable tool for modifying protein functionality [19]. During hydrolysis, hydrophobicity of amino-acid side chains is normally due to relatively small peptides with molecular weights between 1,000 and 6,000 Da. Enzymatic hydrolysis is an effective way of producing bioactive peptides, which are short peptides released from food proteins by hydrolysis and with biological activities that may be beneficial to the organism [20]. Bioactive peptides usually contain 3–20 amino acids per molecule and are inactive within the parent protein molecule sequence [21]. 

The protein hydrolysate obtained from the chia protein-rich fraction had 58.5% ACE-I inhibition. This is lower than the 79.5% reported for yak milk casein hydrolyzed with Alcalase for 240 min [22], but higher than the 5%–50% obtained for protein hydrolysates from amaranth (*Amaranthus hypochondriacus*) albumin 1 and globulin [23].

**Figure 1:**
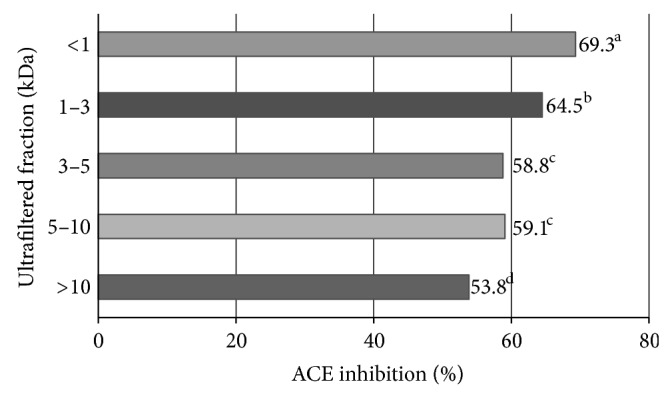
ACE-I inhibition percentage of peptide fractions obtained by ultrafiltration of a *S. hispanica *protein hydrolysate. ^a–d^Different superscript letters indicate statistical difference (*P* < 0.05). Data are the mean of three replicates.

Ultrafiltration of the hydrolysate produced UPFs with increased biological activity. ACE-I inhibition ranged from 53.8% to 69.3% (Figure 1), with clear increases (*P* < 0.05) in activity in progressively smaller fractions; that is, the >10 kDa fraction had the lowest activity and the <1 kDa had the highest. Use of ultrafiltration membranes clearly helped to enrich specific peptide fractions. This coincides with a study in which the <1 kDa fraction of an Alaska pollock (*Theragra chalcogramma*) frame protein hydrolysate had the highest ACE-inhibitory activity (87.6%, IC_50_ = 457 *μ*g/mL) [24]. In another study, protein hydrolysate from Chinese soft-shelled turtle had a lower ACE-I inhibitory effect (IC_50_ = 280 *μ*g/mL) than its corresponding <5000 kDa ultrafiltered fraction (IC_50_ = 190 ± 5 *μ*g/mL) [25]. A greater inhibitory effect at smaller molecular weights was also observed in a yak casein hydrolysate fraction, in which the <6 kDa fraction was the most effective (85.4%) [22]. When taken in conjunction, these results support the suggestion that ultrafiltration is an effective way of enriching ACE-I inhibitory peptides from chia proteins.

### 3.3. Amino Acid Composition

An amino acid profile was generated for the <1 kDa UPF because it had the highest ACE-I inhibitory activity. During hydrolysis, asparagine and glutamine partially converted to aspartic acid and glutamic acid, respectively; the data for asparagine and/or aspartic acid were therefore reported as Asx while those for glutamine and/or glutamic acid were reported as Glx. The high inhibitory activity (69.3%) exhibited by the <1 kDa UPF was probably due to its high concentration of hydrophobic amino acids (41.68 g/100 g), including Pro (6.11 g/100 g), Phe (11.03 g/100 g), Leu (10.23 g/100 g), and Ile (6.57 g/100 g) (Table 1).

Amino acid C-terminal hydrophobicity has the greatest influence on ACE-I inhibitory activity, and the higher the hydrophobicity the higher the inhibitory activity [15]. This coincides with a number of previous studies. Cheung et al. [26] found that dipeptides could have high ACE-I inhibitory activity if C-terminals were aromatic amino acids and proline, and N-terminals were aliphatic amino acid branches. Hellberg et al. [27] measured Cheung's peptides samples in the same laboratory, modeled the results with a QSAR, and found that dipeptides with positively-charged amino acids at the N-terminal and bulky hydrophobic amino acids at the C-terminal had stronger ACE-I inhibitory activity. Using Z descriptors to investigate the quantitative structure-activity relationship of ACE-inhibitory dipeptides, Wu et al. [28] found that ACE-inhibitory activity was strongly affected by the three-dimensional chemical properties and hydrophobicity of C-terminal amino acids; that is, the higher the volume and the greater the hydrophobicity of the amino acids, the higher the ACE-I inhibitory activity. Therefore, some dipeptides with hydrophobic amino acids at the C-terminal, such as phenylalanine, tryptophan, and tyrosine, will have high ACE-I inhibitory activity. Wu et al. [28] also reported that strongly hydrophobic and small N-terminal amino acids, such as valine, leucine, and isoleucine, were more suitable for high-activity tripeptides. Low charge, large size, and weak hydrophobicity in the second amino acid from the N-terminal were more suitable high activity. Finally, for the C-terminal, high charge, large volume, and strongly hydrophobic residues (e.g., aromatic amino acids) were more suitable. In an analysis of ACE-I inhibitory peptides from milk sources, Pripp et al. [29] found that for peptides with ≤6 amino acids at the C-terminal, ACE-I inhibition was strongly affected by hydrophobicity, positive charge, and volume of amino acids adjacent to the C-terminal, whereas the N-terminal amino acid had no direct relationship. Hydrophobicity and C-terminal amino acid size are apparently the principal aspects affecting ACE-inhibitory activity. It stands to reason that hydrophobic amino acids, aromatic amino acids, and branched-chain amino acids are important components in high-activity peptides, and that proteins with high contents of these amino acid types (especially aromatic amino acids) have more potential to produce high activity ACE-I inhibitory peptides. Therefore, digestion of proteins to produce peptides with hydrophobic amino acids at the C-terminal will tend to increase ACE-I inhibitory activity in any derived hydrolysates [20].

**Figure 2:**
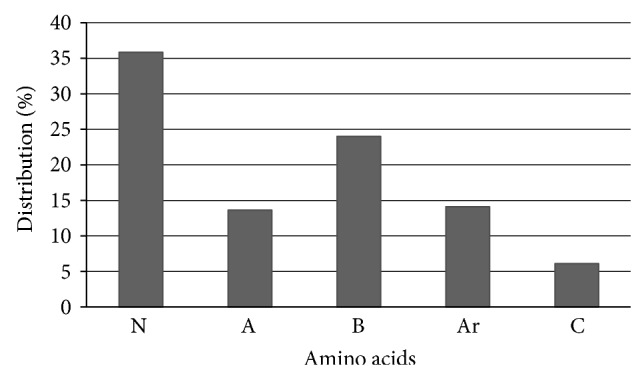
Amino acid distribution in the <1 kDa ultrafiltered fraction of a *S. hispanica* protein hydrolysate. N: neutral amino acids (including Gly, Ala, Ser, Thr, Val, Leu, and Ile). A: acid amino acids (including Asp and Glu). B: basic amino acids (including Lys, His, and Arg). Ar: aromatic amino acids (including Phe, Tyr, and Trp). C: cyclic amino acids (including Pro).

Although the structure-activity relationship of ACE-I inhibitory peptides has not yet been established, these peptides show some common features. Studies of the structure-activity relationships in different ACE-I inhibitory peptides indicate that binding to ACE is strongly influenced by substrate C-terminal tripeptide sequence. Ondetti and Cushman [30] proposed a binding model for interactions between the substrate and active ACE site. C-terminal tripeptide residues may interact with the S1, S1′, and S2′ subsites at the active ACE site. ACE appears to prefer substrates or competitive inhibitors that contain hydrophobic amino acid residues at the three C-terminal positions. Captopril owes its potency and selectivity to chemical design guided by a hypothetical active site model based on the observed properties of ACE and on an analogy to the known active site of a related zinc-containing peptidase. ACE's zinc ion is appropriately located between S1 and S1′, allowing it to participate in hydrolytic cleavage of the substrate peptide bond and resulting in release of a dipeptide product. Studies of the structure-activity relationship in ACE-I inhibitory peptides have shown that those with potent inhibitory activity have proline, phenylalanine, or tyrosine at the C-terminal, as well as hydrophobic amino acids in their sequence [31]. In light of this previous research, the ACE-I inhibitory activity observed here in the chia hydrolysate was probably due to amino acid composition (Figure 2).

### 3.4. Gel Filtration Chromatography of the <1 kDa Ultrafiltered Fraction

**Figure 3:**
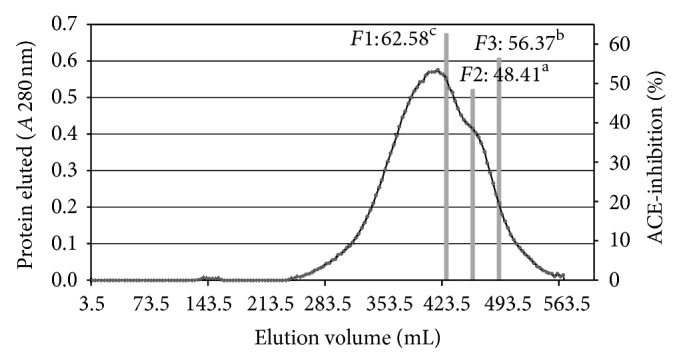
Elution profile of the <1 kDa ultrafiltration fraction of the *S. hispanica* protein hydrolysate purified in a Sephadex G-50 gel filtration column. ^a–e^Different superscript letters indicate statistical difference (*P* < 0.05). Data are the mean of three replicates.

Of the UPFs, the <1 kDa fraction exhibited the highest ACE-I inhibitory activity and was selected for further fractionation. A molecular weight profile was generated of this UPF using gel filtration chromatography (Sephadex G-50 column). This profile was typical of a protein hydrolysate formed by a pool of peptides, with gradually decreasing molecular masses. Elution volumes between 406 and 518 mL included free amino acids and peptides with molecular masses ranging from 0.4 to 3.6 kDa. This range was fractionated into three fractions and ACE-I inhibitory activity determined for each. Fractions with elution volumes smaller than 406 mL and greater than 518 mL were not analyzed because they largely included peptides with high molecular weights, as well as free amino acids. ACE-I inhibitory activity (%) in the <1 kDa UPF ranged from 48.4% to 62.6% (Figure 3). The highest ACE-I inhibitory activity was observed in fraction F1 (62.6%; 427–455 mL elution volume). Its molecular mass was approximately 1.5–2.5 kDa, indicative of 7–12 amino acid residues. The IC_50_ value for F1 (3.97 *μ*g/mL) was lower than those of gel filtration (Sephadex G-25) peptide fractions from tuna broth hydrolysate (210 to 25,260 *μ*g/mL) [32] or from buckwheat *Fagopyrum esculentum* Moench (Sephadex C-25 = 25,715.1 *μ*g/mL; Sephadex G-10 = 21,315.1 *μ*g/mL). Other studies suggest that ACE inhibition by hydrolysates depends on the source species and hydrolysate purity level. For instance, production of a 1–3 kDa protein hydrolysate from Alaska pollock frame using an ultrafiltration membrane bioreactor system resulted in high ACE-I inhibition (IC_50_ = 110 *μ*g/mL), but further purification using consecutive chromatographic methods in a SP-Sephadex C-25 column and HPLC in an octadecylsilane column resulted in a still stronger effect (IC_50_ = 14.7 *μ*g/mL) [24]. In another example, an enzymatic hydrolysate from cuttlefish (*Sepia officinalis*) muscle protein was found to have high ACE-I inhibitory activity (87.1 ± 0.9% at 200 *μ*g/mL). However, size exclusion chromatography with a Sephadex G-25 produced a fraction (P6) with yet higher ACE-I inhibition (IC_50_ = 11.6 *μ*mol/L), which, when fractionated by reverse-phase (RP)-HPLC, was found to contain Ala-His-Ser-Tyr, Gly-Asp-Ala-Pro, Ala-Gly-Ser-Pro, and Asp-Phe-Gly [33].

In ACE-I inhibitory peptides from chia, the protein-rich fractions are not as potent as hypertension treatment drugs but hold promise as a safe, natural therapeutic agent without adverse side effects. The potential of chia protein-derived peptides as antihypertension agents depends on the ability of these peptides to reach their target site without suffering degradation and consequent inactivation by intestinal or plasma peptidases. Resistance to peptidase degradation is a probable prerequisite for any ACE inhibitory hydrolysates/peptides to exercise an antihypertensive effect after oral or intravenous administration. Proline-containing peptides are generally resistant to degradation by digestive enzymes [34], and tripeptides containing a Pro-Pro C-terminal are resistant to proline-specific peptidases [35]. However, peptide degradation or fragmentation results in smaller peptides and therefore in potentially more potent ACE-I inhibitory activity. Clearly, *in vivo* studies are needed to confirm the effect of these peptides since it is both difficult and unwise to extrapolate directly from *in vitro* to *in vivo* activity. The main challenges in doing this are determining bioavailability of ACE-I inhibitory peptides after oral administration and the fact that peptides may influence blood pressure by mechanisms other than ACE-I inhibition. To exert an antihypertensive effect after oral ingestion, ACE-I inhibitory peptides must reach the cardiovascular system in an active form, meaning that they need to remain active during digestion by human proteases and transport through the intestinal wall into the blood. Bioavailability has been studied for some ACE-I inhibitory peptides, and it is known that proline-containing peptides are generally resistant to degradation by digestive enzymes. Peptides can be absorbed intact through the intestine by paracellular and transcellular routes, although postabsorption bioactivity potency is inversely correlated to chain length [36].

All the chia derivatives studied here had IC_50_ values far higher than the 0.0013 *μ*g/mL of Captopril, a synthetic ACE-I inhibitor [32]. Nonetheless, the chia purified peptides have biological potential, and the *F* < 1 kDa fraction had high ACE-I inhibitory activity, suggesting that ACE-I inhibitory peptides are rich in hydrophobic amino acids (aromatic or branched chains) and in proline. ACE-I inhibiting peptides from food sources have garnered increasing attention in recent years as promising natural biofunctional alternatives to synthetic drugs. Many of these peptides have been discovered in enzymatic hydrolysates of different food-source proteins and subsequently applied in the prevention of hypertension and initial treatment of mildly hypertensive individuals. The ongoing search for natural ACE inhibitors may eventually help to create safer and less costly alternatives to synthetic pharmaceutical treatments [25].

## 4. Conclusions

Chia proteins hydrolyzed with the Alcalase-Flavourzyme sequential enzyme system resulted in hydrolysates with ACE-I inhibitory activity. Ultrafiltration produced a very low molecular weight fraction (<1 kDa) which had the highest activity. The hydrolysates and ultrafiltered fractions are potential ingredients for development of functional foods. Enzymatic hydrolysis and ultrafiltration are promising bioprocesses for production of new bioactive food ingredients such as ACE inhibitory peptides purified from chia hydrolysate.

## Figures and Tables

**Table 1:** Amino acid contents (g/100 g) of the <1 kDa ultrafiltered fraction from a *S. hispanica* protein hydrolysate.

Amino acid	*F* < 1 kDa
Asx	7.17
Glx	6.47
Ser	5.14
His	4.04
Gly	4.48
Thr	4.34
Arg	12.36
Ala	3.34
Pro	6.11
Tyr	2.46
Val	1.73
Met	2.06
Cys	4.24
Ile	6.57
Trp	0.61
Leu	10.23
Phe	11.03
Lys	7.61
